# Stokes–Mueller method for comprehensive characterization of coherent terahertz waves

**DOI:** 10.1038/s41598-020-72049-9

**Published:** 2020-09-22

**Authors:** Xin Chai, Xavier Ropagnol, Luis. Sanchez Mora, S. Mohsen Raeiszadeh, Saffiedin Safavi-Naeini, François Blanchard, Tsuneyuki Ozaki

**Affiliations:** 1 INRS-EMT, Advanced Laser Light Source, INRS, Varennes, Québec J3X1S2 Canada; 2 Electrical Engineering Department, École de Technologie Supérieure, Montréal, Québec H3C1K3 Canada; 3 University of Waterloo, Waterloo, ON N2L 3G1 Canada

**Keywords:** Optical spectroscopy, Optics and photonics

## Abstract

Ideally, the full characterization of coherent terahertz (THz) pulses would provide information on the amplitude and direction of its THz electric field, in space and in time, with unlimited dynamic range. Here, we propose and demonstrate a new approach based on the Stokes–Mueller formalism. Our approach can measure the full temporal and spatial variation of coherent THz fields, as well as its polarization state with a high dynamic range. This method employs a simple configuration, using a polarization state analyzer after the electro-optic sampling crystal. This technique could allow high sensitivity due to its ability to use thick detection crystals, which also would lead to improved spectral resolution by allowing longer scans in the time domain.

## Introduction

The widely used electro-optical (EO) sampling technique has made terahertz (THz) time-domain spectroscopy (THz-TDS) an extremely powerful tool to investigate the linear and nonlinear properties of materials in the far-infrared spectral range^1–3^. In EO sampling, a THz field modulates the birefringence of the detection crystal, which changes the polarization state of the probe optical laser pulse^1^. The THz electric field is then retrieved by measuring this change in polarization of the probe beam using a balanced detection configuration^1^.

For linear THz spectroscopy, coherent detection allows one to fully extract the complex refractive index of the material without assuming the Kramers–Kronig relations^4^. Recently, with the development of numerous intense THz sources^5–9^, various ultrafast subcycle nonlinearities have been revealed through straightforward time-domain analysis enabled by EO sampling^10,11^. However, such experiments are challenging due to the small-angle approximation used in the conventional EO sampling technique^1,12,13^. Furthermore, over-rotation may take place when the phase delay $\phi$ surpasses $90^\circ$, where $\sin \phi$ is symmetrical around 90°^14–17^. Consequently, intense THz fields can only be detected by using thin detection crystals with small EO coefficient, or by reducing the THz beam intensity before the detection crystal with multiple attenuators^10,11,18^. These approaches, which are necessary to limit the phase delay within the small-angle approximation, limit the amplitude of the detected signal and as well the dynamic range. Moreover, the spectral resolution is relatively small due to the short scanning window of the temporal trace for avoiding the multi-echo coming from the Fabry-Perrot effect occurring at the interface of each attenuator and the detector crystal. Further, since THz attenuators usually are only used for high-field measurements, it becomes challenging to make a direct spectral comparison between high-field and low-field responses. This difficulty is because to obtain a better signal-to-noise ratio, THz attenuators are not used for low-field THz measurements.

On the other hand, measuring the change in the THz electric field orientation is usually complicated. Several methods have been proposed using both multi-contact photoconductive antenna and conventional EO sampling technique^19–35^. For these experiments involving elliptical or circular THz polarization, one must rotate a wire-grid polarizer manually for the detection of two orthogonal components and to optimize the orientation of the detection crystal^19–23^. To solve this problem, various approaches have been proposed for the measurement of the polarization state of the THz wave^24–31^. However, most of the techniques require the rotation of various optical elements or have only been demonstrated at relatively low THz fields.

Here, we propose and demonstrate a new technique that considers the complete polarization variation of the optical probe beam. To this end, we describe the polarization of light using the more general Stokes–Mueller formalism. As shown in the following discussion, the Stokes vector provides a complete description of the polarization state of an electromagnetic wave. Thus, the measurement of different combinations of Stokes parameters provides different information on the THz waves, leading to a multi-purpose technique for characterizing the THz pulse. Furthermore, this technique is easy to implement, has ultrahigh dynamic range since it is not limited by the small-angle approximation or over-rotation, and can also measure not just the amplitude but the polarization direction of the THz field, all as a function of space and time. Real-time self-referenced imaging of THz electric field vector can also be realized by using a camera to detect the optical probe beam with a spot size larger than that of the THz beam^15^.

## Results and discussion

The Stokes vector represents the full polarization state of light, and the effect of an optical element is described by a $4 \times 4$ Mueller matrix^36^. The Stokes vector of the transmitted light can be calculated by a simple multiplication between the Mueller matrix and the incident Stokes vector. For the THz detection system, the detector crystal can be treated as a rotating waveplate controlled by the THz electric field^12,13,23^. By measuring the Stokes parameters of the probe beam, we can therefore obtain the THz field and polarization information. For general purposes, we develop the technique using the four Stokes parameters here, which eventually allows a self-referenced measurement^15^. In practice, three components are sufficient for the characterization of an intense elliptically polarized THz pulse.

**Figure 1:**
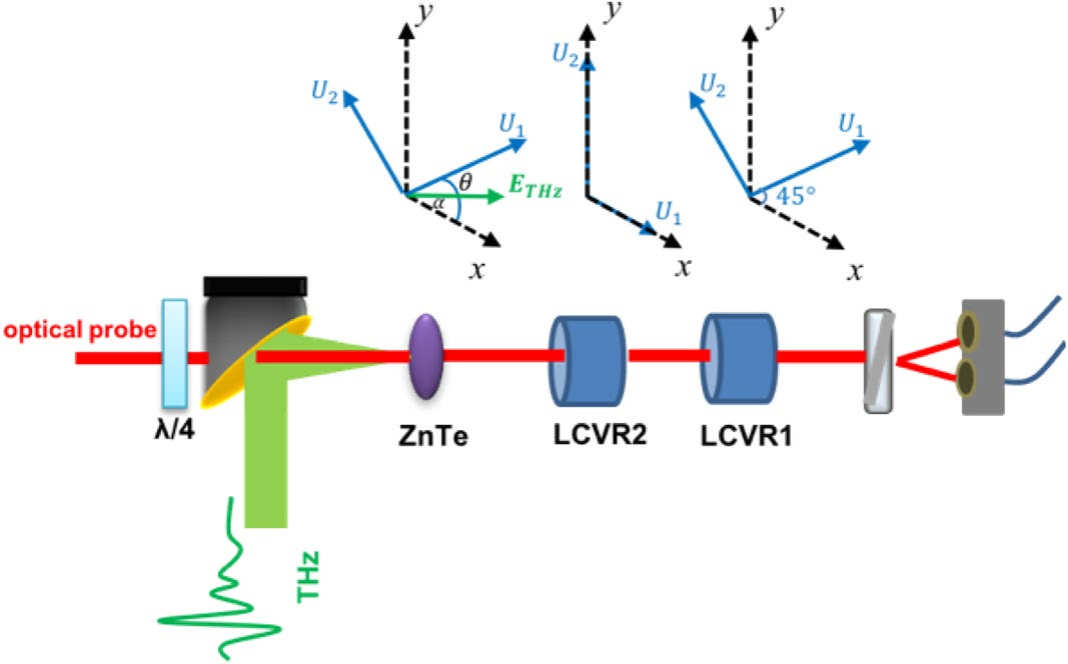
Schematic diagram of the detection system with a polarization state analyzer (PSA), which is composed of two liquid crystal variable retarders (LCVR) and one Wollaston prism. U_1_ and U_2_ are the principal axes of each element.

As shown in Fig. 1, a quarter-wave plate is used to change the optical probe from linear polarization to circularly polarization. If a linearly polarized probe beam is used, when the THz polarization starts to rotate, the induced refractive-index axes may end up along the polarization orientation of the linearly polarized probe beam, giving rise to a false zero amplitude. The Stokes vector of a right circularly polarized probe beam can be described by^32^:

1 $$ S_{in} = \left( {\begin{array}{*{20}c} {\begin{array}{*{20}c} 1 \\ 0 \\ \end{array} } \\ {\begin{array}{*{20}c} 0 \\ 1 \\ \end{array} } \\ \end{array} } \right) $$

Here, we write the Mueller matrix of a rotated waveplate to describe the nonlinear detection crystal^36^, where $\phi$ is the phase retardation induced by the THz field and $\theta$ is the rotation angle of the induced refractive-index axes due to the variation of THz polarization orientation $\alpha$:

2 $$ {\text{M}}_{{{\text{EO}}}} = \left( {\begin{array}{*{20}c} {\begin{array}{*{20}c} 1 & 0 \\ 0 & {\cos^{2} 2\theta + \cos \phi \sin^{2} 2\theta } \\ \end{array} } & {\begin{array}{*{20}c} 0 & 0 \\ {\left( {1 - \cos \phi } \right)\sin 2\theta \cos 2\theta } & {\sin \phi \sin 2\theta } \\ \end{array} } \\ {\begin{array}{*{20}c} 0 & {\left( {1 - \cos \phi } \right)\sin 2\theta \cos 2\theta } \\ 0 & { - \sin \phi \sin 2\theta } \\ \end{array} } & {\begin{array}{*{20}c} {\sin^{2} 2\theta + \cos \phi \cos^{2} 2\theta } & { - \sin \phi \cos 2\theta } \\ {\sin \phi \cos 2\theta } & {\cos \phi } \\ \end{array} } \\ \end{array} } \right) $$

After transmitting through the detection crystal, the Stokes vector becomes:

3 $$ S_{THz} = \left( {\begin{array}{*{20}c} {\begin{array}{*{20}c} {S_{0} } \\ {S_{1} } \\ \end{array} } \\ {\begin{array}{*{20}c} {S_{2} } \\ {S_{3} } \\ \end{array} } \\ \end{array} } \right) = \left( {\begin{array}{*{20}c} {\begin{array}{*{20}c} 1 \\ {\sin \phi \sin 2\theta } \\ \end{array} } \\ {\begin{array}{*{20}c} { - \sin \phi \cos 2\theta } \\ {\cos \phi } \\ \end{array} } \\ \end{array} } \right) = M_{EO} *S_{in} $$

Here, $M_{EO}$, representing the detection crystal, is the widely used Mueller matrix of a rotated waveplate^36^. For a commonly used (110)-orientated ZnTe crystal, the x-axis of the laboratory frame is along the [− 110]. The delay phase $\phi$ for a (110) zinc blend nonlinear crystal such as ZnTe can then be described by^25,37^:

4 $$ \phi = \frac{{\omega_{0} d}}{2c}n_{0}^{3} r_{41} E_{THz} \sqrt {1 + 3\cos^{2} \alpha } $$

Furthermore, the orientation $\theta$ of the refractive-index axis can be calculated by^25,37^:

5 $$ \cos 2\theta = \sin \alpha /\sqrt {1 + 3\cos^{2} \alpha } $$

Here, ${\varvec{\omega}}_{0}$ is the angular frequency of the optical probe beam, $d$ is the thickness of the detection crystal, $c$ is the speed of light, $\alpha$ is the angle between the THz electric field vector and the [-110] of the ZnTe crystal, $n_{0}$ is the refractive index, and $r_{41}$ is the EO coefficient of the detection crystal. Therefore, by measuring the four elements in $S_{THz}$ of Eq. (3), we obtain the phase delay $\phi$ and the orientation of the refractive-index axis $\theta$. Then the THz electric field $E_{THz}$ amplitude, as well as its polarization orientation, can be retrieved from Eqs. (4) and (5). Experimentally, such measurements can be realized by using the technique of Stokes–Mueller polarimetry^31^ by using a polarization state analyzer (PSA) that includes two liquid crystal variable retarders (LCVR) and one Wollaston prism for balanced detection.

Since only the total intensity $S_{0}$ is observable in the Stokes vector, four intensity measurements are needed, and a $4 \times 4$ matrix $M_{measure}$ is constructed by the first rows of four different Mueller matrices generated by the PSA^36,38,39^. Since the phase delays, as well as the orientation of the LCVR, are known, the $S_{THz} { }$ can be obtained directly from a matrix inversion using the following equation:

6 $$ \left( {\begin{array}{*{20}c} {\begin{array}{*{20}c} {I_{0} } \\ {I_{1} } \\ \end{array} } \\ {\begin{array}{*{20}c} {I_{2} } \\ {I_{3} } \\ \end{array} } \\ \end{array} } \right) = M_{measure} * S_{THz} $$

Here, to minimize the condition number of this matrix to perform the inverse calculation, we use two LCVRs to generate four independent Stokes vectors that can form a regular tetrahedron on the Poincaré sphere^38,39^. The orientations of the principal axis are chosen to be 45° and 0° for LCVR1 and LCVR2, respectively. The four groups of phase delays we used for LCVR1 and LCVR2 are (91.4°, 92.3°), (− 20, 108°), (207.6°, 126°) and (63.3°, − 19.6°), which then generate the measurement matrix $M_{measure}$. This combination of phase delays can provide a minimum condition number of $\sqrt 2$. Since a matrix inverse calculation is needed in Eq. (6), using a low condition number is vital to reduce the impact on the results induced by any experimental errors. Other combinations exist as well and can be used as long as the obtained condition number is minimized ^36,38,39^. To preserve a good signal-to-noise ratio (SNR), we use a balanced detection configuration as the conventional EO sampling technique, and a lock-in amplifier measures the differential signal. As a result, the first element $S_{0}$ of the obtained differential vector $S_{THz}$ becomes zero and $S_{3}$ is proportional to the change of ${\cos}\phi$ away from 1 as $\phi = 0^\circ$.

To demonstrate our new technique under different experimental conditions, we tested it with three different photoconductive antenna (PCA) THz sources: (i) a conventional THz source pumped by a femtosecond oscillator, (ii) a relatively intense THz source with linear polarization ($\approx 65\;{\text{ kV}}/{\text{cm}}$) and (iii) an intense quarter-cycle elliptically polarized THz source^40^. We used the same 1 mm (110) ZnTe detection crystal for all the experiments.

**Figure 2:**
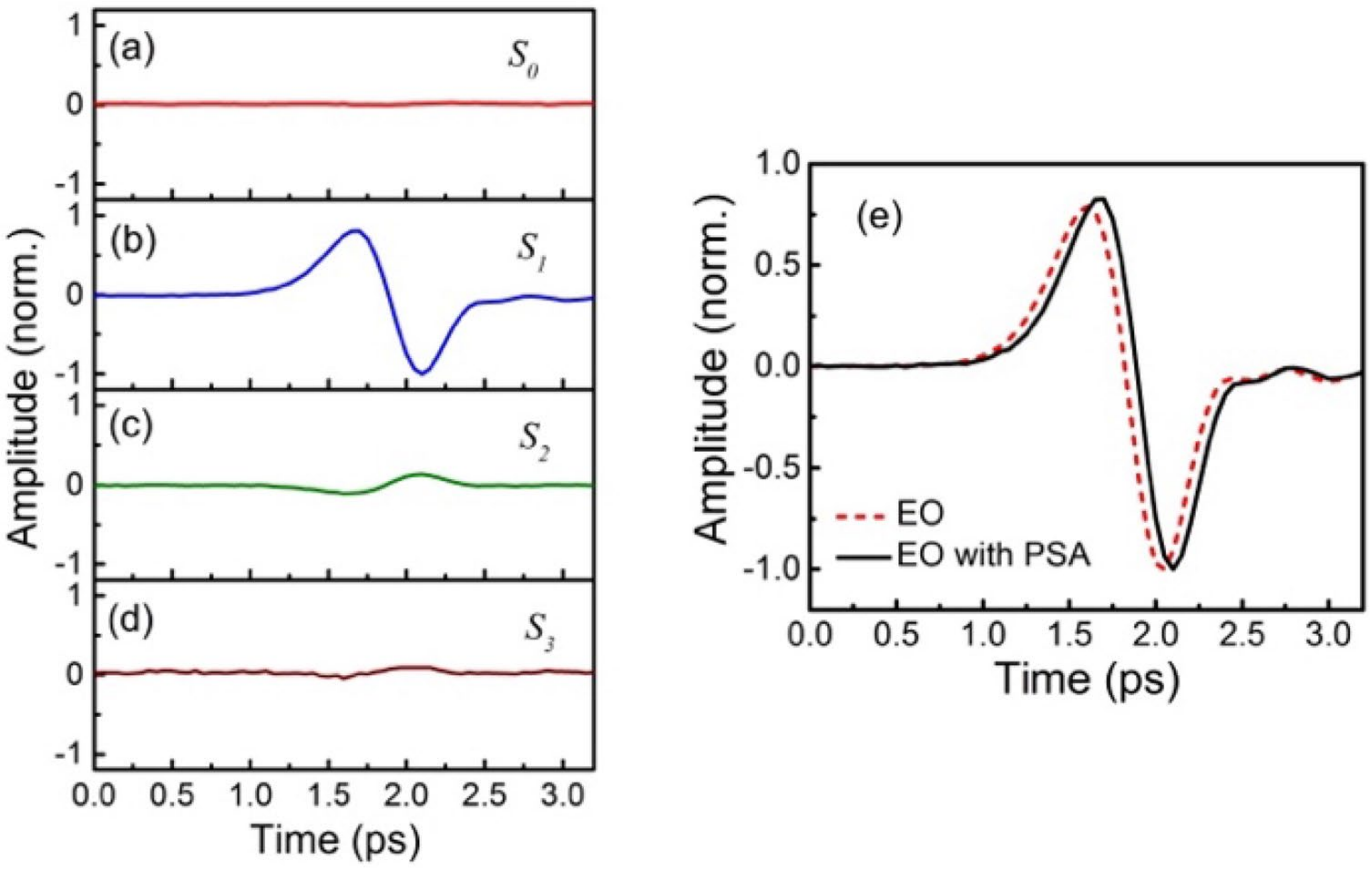
Temporal Stokes parameters of the balanced probe signal (**a**)$$ S_{0}$$, (**b**) $$S_{1}$$, (**c**) $$S_{2}$$, and (**d**) $$S_{3}$$. (**e**) The extracted THz signals by using the system with a PSA and by using the conventional EO sampling technique.

Figure 2a–d shows the measured four Stokes parameters $S_{0}$, $S_{1}$, $S_{2}$ and $S_{3}$ using the conventional THz source pumped by a femtosecond oscillator. As expected, a signal with approximately 0 amplitude is observed for $S_{0}$. The THz induced birefringence is then measured by $S_{1}$.

As shown in Fig. 2e, the obtained THz curve from EO sampling is similar to the measured ${\sin}\phi$ of $S_{1}$, which is in accordance with the small-angle approximation^1,12,13^. Here, a weak signal with the opposite polarity of $S_{1}$ is observed on $S_{2}$. This effect can be induced by the orientation discrepancy between the THz polarization orientation and the crystallographic axis of ZnTe. As can be seen from Eq. (3), since the THz polarization orientation does not change, this experimental error can be easily removed by comparing the amplitude peak of $S_{1}$ and $S_{2}$. Here, the variation of ${\cos}\phi$ from 1 is measured by $S_{3}$. Since the cosine function varies slowly at small angles, the amplitude of $S_{3}$ is relatively small and close to the noise reference level, as is $S_{0}$. By comparing the amplitude of $S_{1}$ and $S_{3}$ at 2.2 ps, we obtain a peak electric field of less than 1 kV/cm. Even though this value can only be used as a rough estimate due to the limitation of the SNR of $S_{3}$, this direct measurement of THz electric field is not accessible here by using other techniques such as conventional EO sampling when the electric field is low. As can be seen from the comparison between the obtained THz signals in Fig. 2e, our technique can measure oscillator-pumped weak THz pulses equally well as conventional EO sampling techniques.

**Figure 3:**
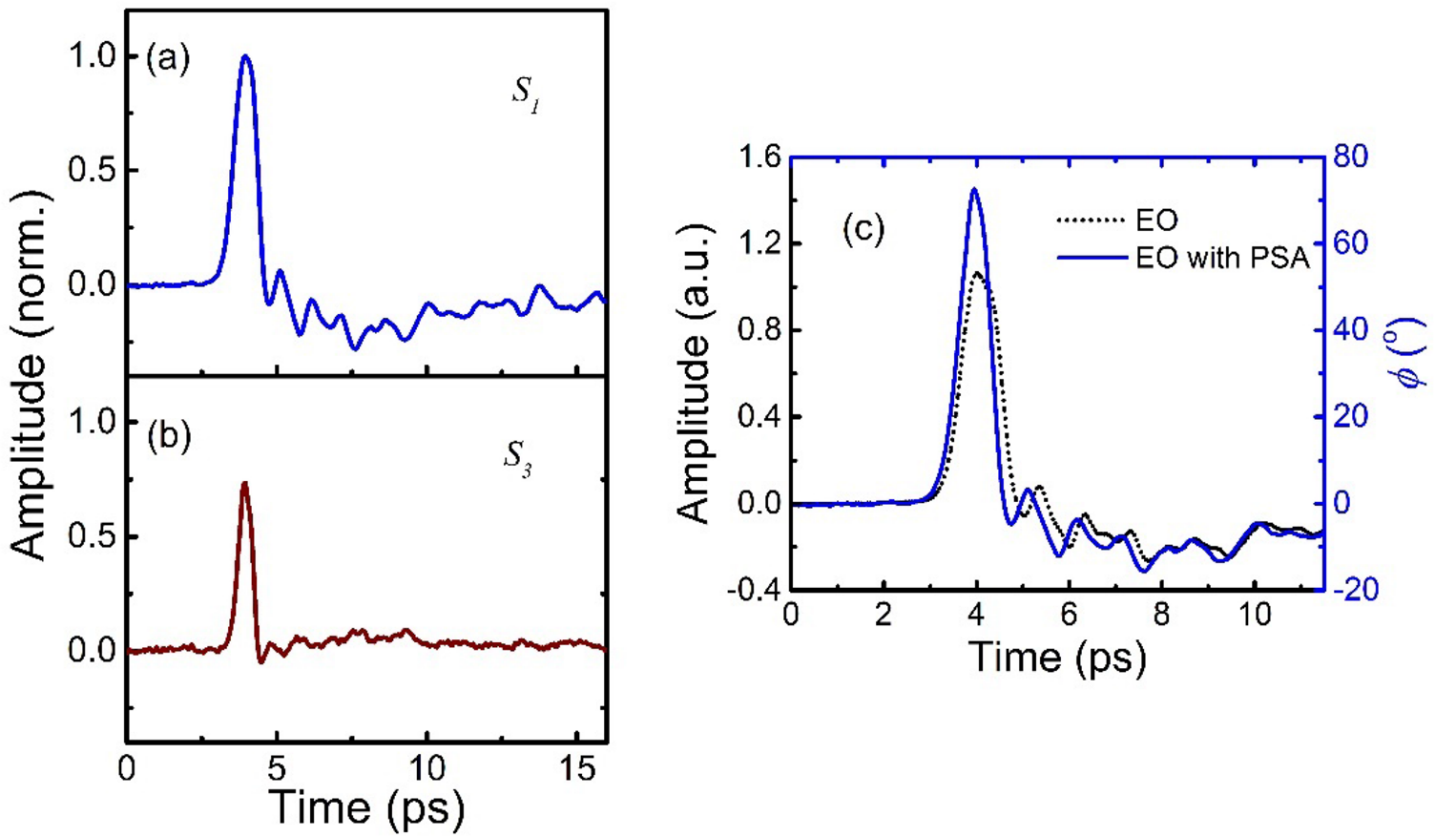
Temporal Stokes parameters of the balanced probe signal (**a**) $S_{1}$, and (**b**) $S_{3}$. (**c**) Measured THz signals by using the system with a PSA and by using the conventional EO sampling technique. Here, we show the measured phase delay induced by the THz electric field for the results obtained using our PSA system.

Next, we tested our technique in a THz-TDS system with an intense THz source. Here, a specially designed interdigitated large-aperture photoconductive antenna (ILAPCA) THz source is used. Figure 3 shows the experimental results using our technique, and a comparison with the EO sampling technique. Here, the measured signals of $S_{1}$ and $S_{3} { }$ are sufficient to reveal the intense THz signal with a high dynamic range. The ultimate limitation will be the detection crystal itself^42,43^, in which atomically strong electric fields may lead to the generation of charge carriers via Zener tunneling and, in turn, completely change the detection probe signal^42^. Further, the THz Kerr effect may take place at high fields, which may eventually distort the detection signal as well ^43^. As shown in Fig. 3a,b, the low-field and high-field details are retrieved precisely from $S_{1}$ and $S_{3}$, respectively. On the other hand, in Fig. 3c, we can see the difference between the traces obtained by a standard EO sampling detection and our technique. The principal difference is the difference in amplitude and the compression of the pulse in time. Both differences are attributed to the deviation of the small-angle approximation condition, which must be fulfilled for the standard EO sampling detection^1,12,13^. Moreover, higher fields may cause the problem of over-rotation for EO sampling, which eventually limits the maximum detection range to quarter-cycle phase retardation^14–16^. In our new technique, by measuring ${\cos}\phi$ simultaneously, we can calculate directly the peak phase retardation $\phi$, which is approximately 72°, which is equivalent to an electric field of 64.3 kV/cm. To verify this value, we measured the modulation at the peak position for the two photodetectors by performing conventional EO sampling using a 300 μm (110) GaP crystal and obtain a field strength of 61 kV/cm^1^.

To demonstrate the THz-polarization sensitive detection, we then removed the wire-grid polarizer and performed the experiment using our detection system. To generate an elliptically polarized THz pulse, we used a delay mask with a thickness of 0.12 mm, which leads to a time delay of approximately 200 fs between the peaks of the horizontal ($E_{x}$) and the vertical ($E_{y}$) THz waveforms. Here, the conventional EO sampling technique is no longer applicable in a single measurement limit, and the 3D THz waveforms can only be obtained by measuring the polarization variation of the probe beam via Jones calculus^25,27,37^ or Stokes–Mueller calculus used here.

**Figure 4:**
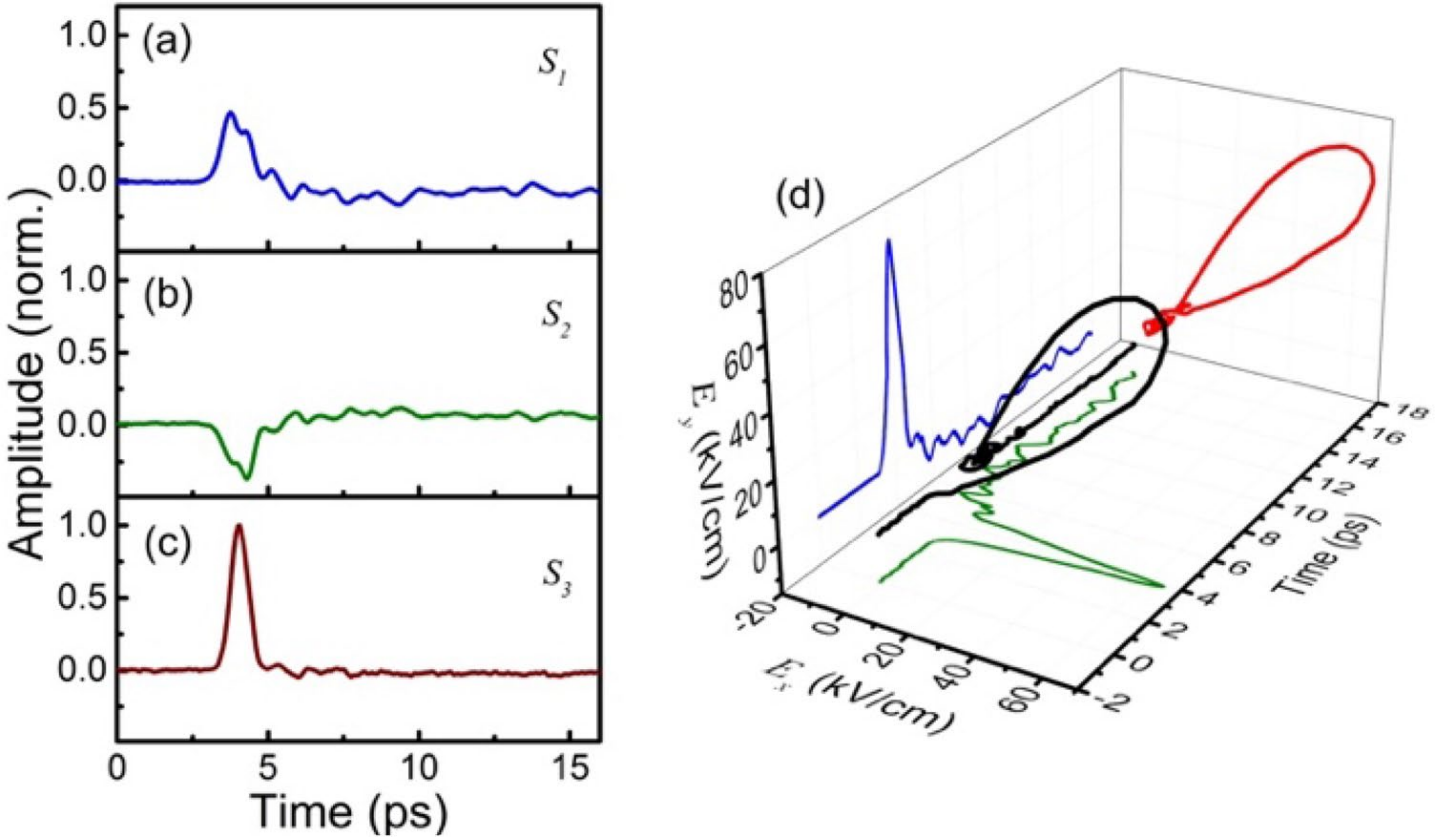
Temporal Stokes parameters of the balanced probe signal (**a**) $S_{1}$, (**b**) $S_{2}$, and (**c**) $S_{3}$. (**d**) Measured 3D THz waveform obtained from $S_{THz}$.

In Fig. 4, we show the measured three Stokes parameters $S_{1}$, $S_{2}$ and $S_{3}$. In practice, $S_{1}$ and $S_{2}$ are sufficient for the detection of elliptically polarized THz pulses with low fields. However, at relatively high fields, it is also necessary to measure $S_{3}$, because the variation of polarization may affect similarly on the signal of ${\sin}\phi$ as that from saturation or over-rotation. The rotation of the refractive-index axis $\theta$ is calculated from $S_{1}$ and $S_{2}$, and then the THz field orientation $\alpha$ can be obtained by using Eq. (4). Figure 4d shows the measured 3D THz waveform, where the maximum is reached at 4.05 ps, corresponding to an electric field of 89.5 kV/cm with a polarization orientation $\alpha$ of 54°. Here, the field-induced phase delay $\phi$ already exceeds 90° and as a result, the correct value of $\phi$ can only be retrieved by measuring both $S_{1}$ and $S_{3}$, because ${\sin}\phi$ starts to decrease for over-rotated $\phi$ above 90°.

As demonstrated by the three measurements, one of the advantages of the current system is that it can be used for different experimental purposes. When using a lock-in amplifier, the measurement of $$S_{0}$$ is not necessary. However, it can always be used as a reference signal, which eventually allows a self-referenced THz measurement by using thicker crystals that can increase the amplitude of the detected THz signal^15,16^. By measuring both $$S_{1}$$$$\left( {\cos \phi } \right)$$ and $$S_{3}$$$$\left( {\sin \phi } \right)$$, the over-rotation problem that exists in the conventional EO sampling technique is solved naturally. Furthermore, the same sensitivity is preserved for both low and high fields, because high signal gains are alternatively provided by sine and cosine signals measured by $$S_{1}$$ and $$S_{3}$$. By adding the information of $${ }S_{2}$$, we can measure the THz polarization variation as well.

## Conclusion

In summary, we have proposed and demonstrated a new EO sampling configuration that is simple and effective for the coherent detection of intense THz waves with any polarization state. The novel voltage controllable polarization modulators, such as LCVR, enables a complete polarization measurement of the probe beam without mechanical rotation of any optical elements. Moreover, our technique can operate under multiple modes to fulfill different experimental requirements. Our detection approach can also be used in future THz imaging applications and studies of anisotropic materials^26,44,45^.

## Methods

For the first experiment of linearly polarized THz wave detection, the THz pulse is generated by a PCA with silver paint electrodes (600 ${\mu m}$ gap size), which is pumped by a 70 fs, 0.5 W Ti: Sapphire oscillator laser. The applied DC voltage on the antenna is 50 V.

This ILAPCA used in our experiment is composed of two sets of electrodes that are perpendicular to each other, which allows the generation of THz electric fields with crossed polarization. By using a delay mask for one polarization^40,41^, we can artificially generate a right-hand or left-hand elliptically polarized quarter-cycle THz pulses. The ILAPCA is pumped by a 15 mJ, 60 fs, 400 nm wavelength laser beam. Since the ILAPCA generates THz pulses with elliptical polarization, and for testing our new detection technique in different conditions, we placed a wire grid polarizer in the THz beam path, allowing only one polarization, for detection of THz pulse with linear polarization.

The PSA is composed of two LCVRs (LCR100, Meadowlarks Optics) and one Wollaston prism. Then two balanced photodiodes are used to measure the intensity difference. Both LCVRs have been calibrated for the wavelength of the optical probe beam (800 nm).

Based on the Stokes–Mueller formalism and the equations between the phase delay and the terahertz electric field, we developed a program that controls the LCVR and performs the conversion from the measured intensity difference to the phase delay as well as the THz electric field. When the phase delay is over 360°, our program can automatically unwrap the phase and provide the correct THz amplitude.
